# Metabolic Profiles of Critically Ill COVID-19 Patients: A Comparative Analysis of Energy Expenditure

**DOI:** 10.7759/cureus.70980

**Published:** 2024-10-07

**Authors:** Rita P Medeiros, Ricardo Sousa Marinho, Inês Magalhães, Maria Ruão, Marisa Cunha, Eduarda Leitão, Rita Costa, Daniela Neto, Álvaro Moreira da Silva, Aníbal Sousa Marinho

**Affiliations:** 1 Intensive Care Unit, Centro Hospitalar Universitário do Porto, Porto, PRT; 2 Intensive Care Unit, Instituto Ciências Biomédicas Abel Salazar, Porto, PRT; 3 Intensive Care Unit, Centro Hospitalar Universitário de Santo António, Porto, PRT

**Keywords:** catabolic stress, covid-19, energy expenditure, indirect calorimetry, metabolic requirements, nutrition and metabolism, nutrition in critical care

## Abstract

Introduction and aims

The COVID-19 pandemic has placed a significant burden on healthcare systems worldwide, especially in the management of critically ill patients. Accurate assessment of energy expenditure (EE) is crucial for providing optimal nutritional support and improving clinical outcomes in these patients. Indirect calorimetry (IC) is the preferred method for measuring EE, yet its use in critically ill COVID-19 patients has not been extensively documented. This study aims to evaluate EE in critically ill patients with and without COVID-19 to identify differences that may impact nutritional support strategies.

Methods

We conducted a retrospective observational study at a large tertiary university hospital, focusing on critically ill patients. The study compared calorimetry data between patients with and without COVID-19. Data collected included resting energy expenditure, energy expenditure per kilogram, and respiratory exchange rate quotient using IC. Statistical analysis was conducted using SPSS Version 28, with a significance level set at p < 0.05.

Results

The study included 95 critically ill patients, comprising 61 non-COVID-19 patients and 34 COVID-19 patients. It was found that COVID-19 patients had significantly lower Simplified Acute Physiology Score (SAPS) II compared to their non-COVID-19 counterparts. Although no significant differences in energy needs over time were detected between the two groups or within the COVID-19 group alone, there was a trend towards lower energy needs in COVID-19 patients. Significant differences were noted in respiratory quotient among COVID-19 patients, particularly between those with and without pulmonary thromboembolism, with the latter group exhibiting lower RQ values.

Conclusion

Our study suggests that while overall EE may not significantly differ between critically ill COVID-19 and non-COVID-19 patients, there are potential differences in substrate use that underline the necessity for individualized nutritional support. These findings highlight the importance of tailoring nutritional interventions to meet the specific needs of critically ill patients, particularly those with COVID-19.

## Introduction

The coronavirus disease 2019 (COVID-19) pandemic has presented unprecedented challenges to healthcare systems worldwide, particularly in the management of critically ill patients presenting with severe respiratory distress, multi-organ dysfunction, and metabolic alterations [1]. Patients with COVID-19 often require prolonged mechanical ventilation (MV) and admission to an intensive care unit (ICU), leading to immobility, catabolic stress, and muscle wastage [2]. As with any critically ill patient, optimal nutritional support is crucial for recovery and improved outcomes [3]. However, the pathophysiology of COVID-19, based on an ambient of hyperinflammation and cytokine storm, may introduce distinct metabolic alterations, which warrants specific investigation [4,5].

Optimal nutritional support is essential in managing COVID-19, especially for patients at risk of malnutrition, both during the acute phase of illness and throughout recovery [5,6]. Poor nutritional status increases the length of hospital stay and poorer clinical outcomes [5]. However, determining appropriate nutritional needs for these patients remains challenging due to the complex interplay of factors affecting their metabolic state, including hyperinflammation and cytokine storm induced by SARS-CoV-2 infection, which introduces distinct metabolic changes, such as increased energy expenditure (EE), altered protein and lipid metabolism, and organ-specific metabolic dysfunction [4]. Accurate assessment of EE is crucial for optimizing nutritional support and improving clinical outcomes [7]. Indirect calorimetry (IC) provides the gold standard for measuring EE, yet data on its use in critically ill patients with COVID-19 remains limited [8-10]. Understanding the metabolic profile of critically ill COVID-19 patients in comparison to their non-COVID-19 counterparts is essential for tailoring nutritional interventions and potentially improving patient outcomes. This study aims to address this knowledge gap by comparing calorimetry data between these two groups.

## Materials and methods

Study design and setting

This single-center retrospective observational study was conducted at a large tertiary university hospital. It involved the analysis of prospectively collected data from ICU patients between January 2020 and March 2022. As a single-center study, results may not be generalizable to other settings or patient populations.

Participants and sample size

The study included patients aged ≥18 years admitted to the ICU, consisting of 95 patients in total (61 without COVID-19 and 34 with COVID-19). Due to COVID-19 restrictions and the difficulties associated with using IC during the pandemic, the sample size was limited. This retrospective analysis included all ICU patients for whom IC was used between January 2020 and March 2022. As the number of COVID-19 cases decreased after this period, the opportunity to include more COVID-19 patients diminished. Additionally, many patients were excluded in the initial phase due to elevated needs for FiO2, which precluded safe IC measurements. Consequently, the sample size reflects the entirety of available and eligible patients during the study timeframe.

Exclusion criteria

Exclusion criteria comprised incomplete data records, refusal to participate from the patient or surrogate decision-maker, and clinical instability precluding safe measurement by IC.

Data collection

Data extracted from medical records included age, gender, body mass index (BMI), Sequential Organ Failure Assessment (SOFA) and Simplified Acute Physiology Score (SAPS) II scores at ICU admission, ICU hospitalization duration, ventilation mode, corticosteroid and muscle blocker use, and the presence of pulmonary thromboembolism (PTE).

Measurement and bias considerations

IC (using Q-NRG+, Cosmed) was applied to determine resting energy expenditure (REE) and respiratory exchange ratio (RER) under clinically stable conditions, with normal arterial blood gas pH and FiO_2_ below 70% during ≥15 minutes of steady state. At least one measurement per week was performed for each intubated patient, and data on hemodynamic support, sedation, neuromuscular blockade, ventilatory mode, and prone positioning were collected.

A significant methodological bias arises from using pre-recorded data for anthropometric measurements such as BMI, not directly measured by investigators, introducing potential errors affecting EE analysis and obesity-related interpretations. Moreover, the absence of data on actual nutritional intake is a limitation, as this could influence the results.

Nutritional assessment

Body weight was used for non-obese calculations (BMI < 30), whereas both actual and adjusted body weight were used for obese subjects (BMI > 30). Predicted EE was calculated using the Harris-Benedict equation coinciding with calorimetry measurement points.

Statistical analysis

Data analysis was conducted using SPSS Version 28.0 for Windows (IBM Corp., Armonk, NY), with significance set at p < 0.05. The Shapiro-Wilk test was used to assess data normality. Frequency analysis for qualitative data and descriptive statistics (mean, standard deviation, median, and interquartile range) based on normality verification characterized the sample. To compare groups, the t-test or Mann-Whitney test was employed, depending on normality assumptions. The chi-square or Fisher’s exact test was used to assess associations between qualitative variables. A mixed repeated measures ANOVA was used to evaluate energy needs over time in relation to COVID-19 status, validating normality of residuals. Multiple linear regression with stepwise variable selection assessed BMI, ventilation mode, MV duration, muscle block, and SAPS/SOFA scores influence on energy needs, confirming Gauss-Markov conditions and non-multicollinearity through tolerance coefficients > 0.1.

Ethical considerations

The study was approved by the local ethics committee, and all procedures followed ethical guidelines, including the Declaration of Helsinki. Participant confidentiality and anonymity were maintained throughout, with data de-identified and securely stored, accessible only by co-researchers.

## Results

In this unicentric study, 95 critically ill patients were included (61 patients without COVID-19 and 34 with COVID-19). Characteristics of the study sample are depicted in Table 1. Overall, 71.6% of the study subjects were male, with a mean age of 61.24±12.66 years. Mean BMI was 29.88±5.9.

**Table 1:** Characterization of patients with and without COVID-19 and comparison between the two groups ^a^The t-test for two independent samples. ^b^Chi-square test. ^c^Mann-Whitney test. ^d^Fisher’s exact test. BMI, body mass index; ICU, intensive care unit; PC, pressure control mode; PTE, pulmonary thromboembolism; SAPS, Simplified Acute Physiology Score; SOFA, Sequential Organ Failure Assessment; VC, volume control mode

		COVID		p-Value
		No (n=61)	Yes (n=34)	
Age, mean ± SD		58.18±15.36	61.24±12.66	0.326^a^
Sex, n (%)	Female	18 (66.7%)	9 (33.3%)	0.753^b^
	Male	43 (63.2%)	25 (36.8%)	
BMI, mean ± SD		28.30±8.74	29.88±5.93	0.350^a^
Days of hospitalization in the ICU, median (Q25%–Q75%)		13.00 (7.00–25.00)	24.50 (17.00–31.00)	<0.0001^c^
Death in the ICU, n (%)		14 (56.0%)	11 (44.0%)	0.318^b^
SAPS II		52.25±14.32	42.09±8.32	<0.0001^a^
SAPS II, mean ± SD		8.51±2.66	7.88±2.04	0.237^a^
SOFA, mean ± SD		4 (33.3%)	8 (66.7)	0.024^d^
PTE, n (%)		11 (25.6%)	32 (74.4%)	<0.0001^b^
Ventilation mode, n (%)	VC	49 (61.3%)	31 (38.8%)	0.164^b^
	PC	12 (80.0%)	3 (20.0%)	
Corticoids, n (%)		11 (25.6%)	32 (74.4%)	<0.0001^b^
Muscle lock, n (%)		13 (59.1%)	9 (40.9%)	0.568^b^

As shown in Table 1, the two groups were identical in terms of age, sex, BMI, death in the ICU, SOFA score, ventilation mode, and muscle lock (p>0.05). Regarding the days of hospitalization in the ICU, those who had COVID-19 had a significantly higher number of days of hospitalization (p<0.0001). Regarding the SAPS score, patients with COVID-19 presented significantly lower values (p<0.0001). Regarding PTE, it was found that patients without COVID-19 had a greater tendency not to have it, while those who had COVID-19 had a tendency to have PTE (0.024). Concerning the use of corticosteroids, the same trend was observed, that is, patients without COVID-19 showed a greater tendency not to take them, while those with COVID-19 tended to take them (p<0.0001).

**Figure 1:**
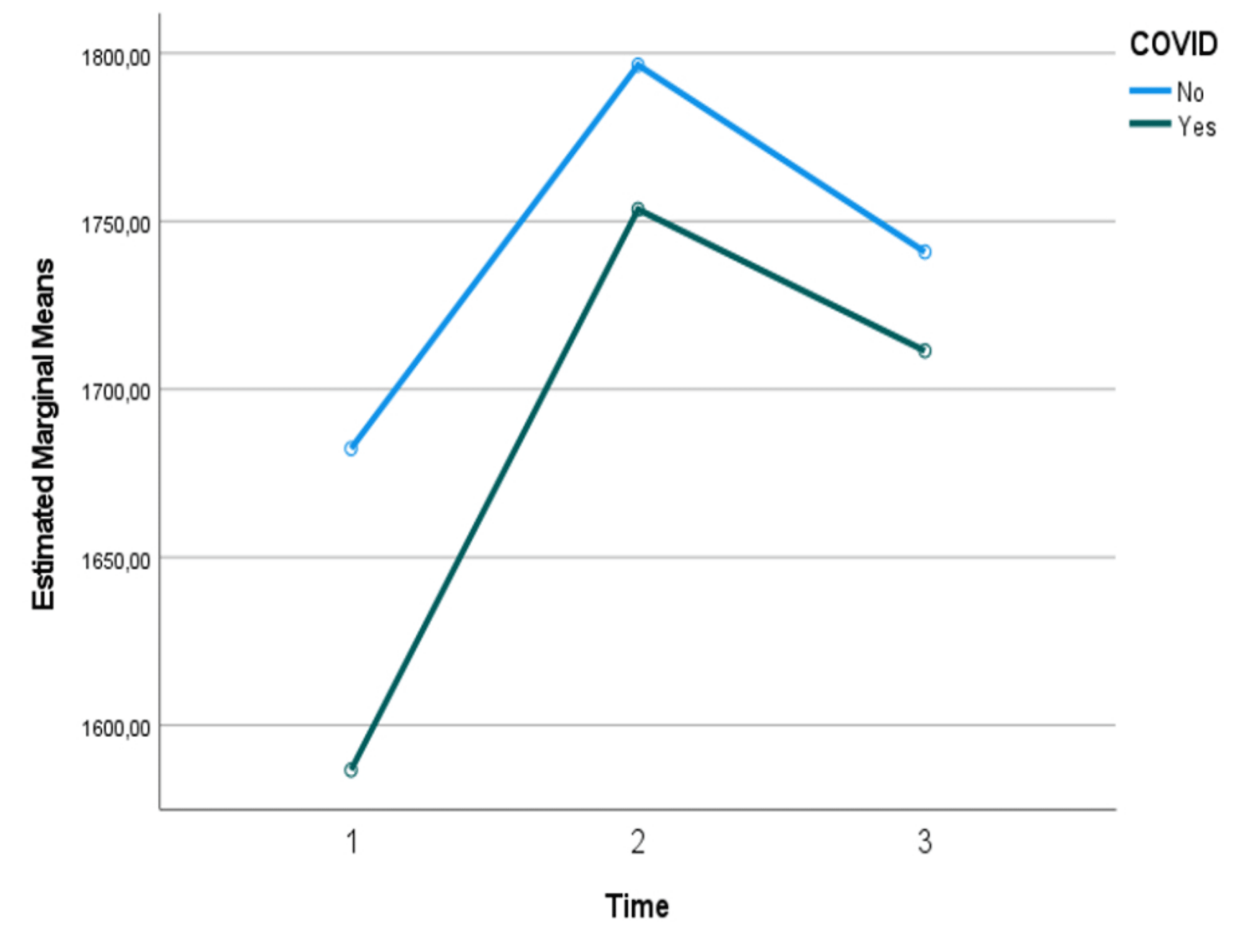
Assessment of resting energy needs over time and considering the presence or absence of COVID-19: results of the mixed repeated measures ANOVA

**Figure 2:**
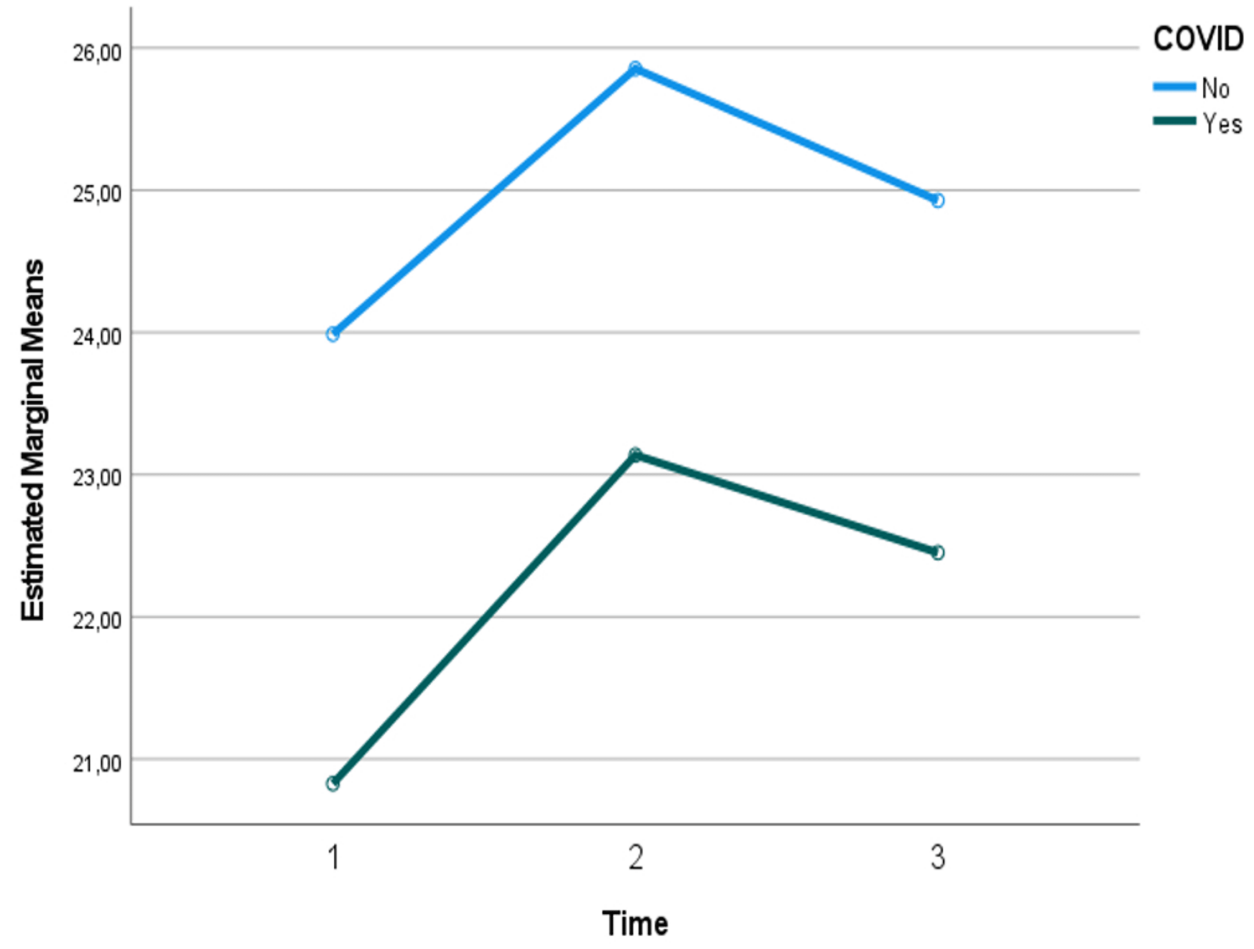
Assessment of energy needs by adjusted weight over time and considering the presence or absence of COVID-19: results of the mixed repeated measures ANOVA

**Figure 3:**
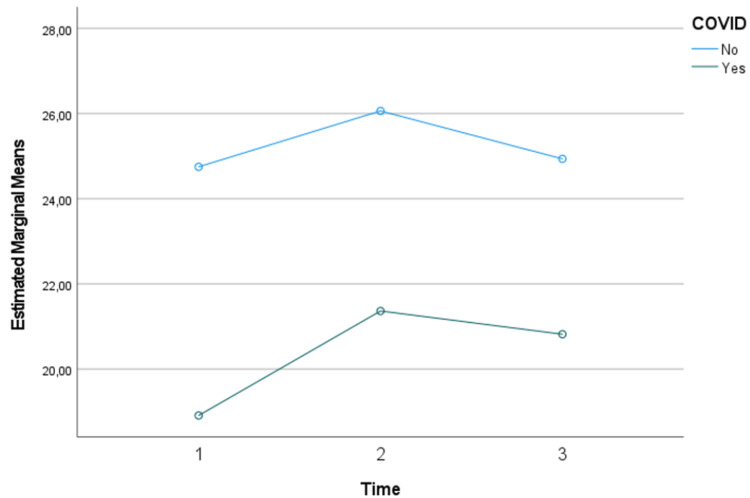
Assessment of energy needs by actual weight over time and considering the presence or absence of COVID-19: results of the mixed repeated measures ANOVA

Regarding the analysis of resting energy needs (REE) by adjusted weight and energy needs (EE) by actual weight over time, when comparing the two groups (COVID-19 vs non COVID-19 patients), no significant changes were detected over time (p>0.05), nor between those who had and those who did not have COVID-19 (p>0.05). However, although not significant, it can be seen that energy needs are lower in those with COVID-19 (Figures 1-3).

**Figure 4:**
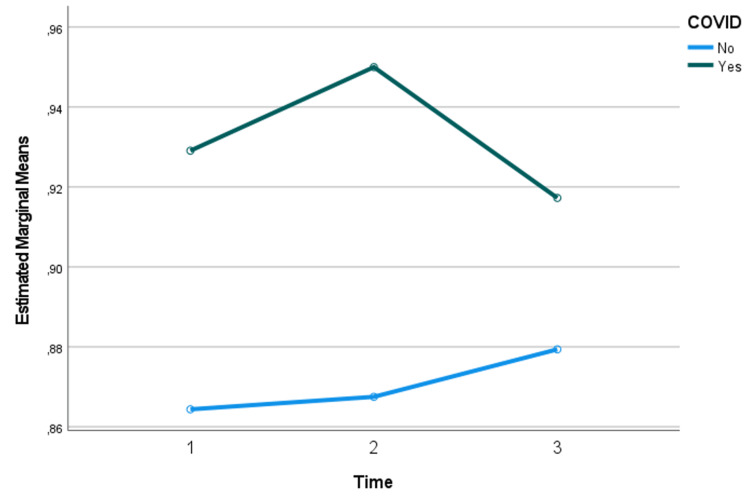
Assessment of respiratory coefficient over time and considering the presence or absence of COVID-19: results of the mixed repeated measures ANOVA

With regard to the respiratory coefficient, no significant changes were detected over time; however, it differed significantly between COVID-19 and non-COVID-19 patients. It was found that the values were significantly higher in those who had COVID-19 (Figure 4).

BMI, ventilation mode, and muscle block (model 3) were identified as predictors of energy needs. As expected, for each additional kg/m^2^, energy needs increase by an average of 15.308 kcal. The pressure-assisted (PA) ventilation mode causes an increase of an average of 293.705 kcal in energy needs. The presence of muscle blockers causes an increase, on average, of 189.028 kcal in energy needs (Table 2).

**Table 2:** Assessment of the influence of BMI, ventilation mode, number of days of mechanical ventilation, muscle block, and SAPS and SOFA scores on energy needs: results of multiple linear regression analysis with the stepwise method Excluded variables in model 1: ventilation mode; muscle block, days of hospitalization in the ICU, and SAPS and SOFA scores. Excluded variables in model 3: days of hospitalization in the ICU, and SAPS and SOFA scores. Excluded variables in model 2: muscle block, days of hospitalization in the ICU, and SAPS and SOFA scores BMI, body mass index; ICU, intensive care unit; SAPS, Simplified Acute Physiology Score; SOFA, Sequential Organ Failure Assessment

Model	Predictors in the model	Unstandardized coefficients	t-Value	p-Value	95.0% confidence interval for B		Collinearity statistics
		B			Lower bound	Upper bound	Tolerance
1	Constant	1177.241	8.401	0.000	898.982	1455.500	
	BMI	16.114	3.439	0.001	6.808	25.420	1.000
2	Constant	1128.223	8.230	0.000	855.961	1400.485	
	BMI	16.405	3.610	0.000	7.381	25.429	0.999
	Ventilation mode	257.256	2.642	0.010	63.902	450.610	0.999
3	Constant	1110.357	8.262	0.000	843.404	1377.310	
	BMI	15.308	3.422	0.001	6.422	24.194	0.988
	Ventilation mode	293.705	3.039	0.003	101.735	485.675	0.971
	Muscle block	189.028	2.249	0.027	22.095	355.961	0.960

Considering only COVID-19 patients, no statistically significant differences were detected regarding energy needs between those who had and those who did not have PTE (p>0.05) (Table 3). However, although not significant, it can be seen that those who had PTE had, on average, greater energy needs. Within the COVID-19 group alone, the respiratory quotient (RQ) differed significantly between patients with and without PTE. Patients without PTE exhibited lower RQ values (t(32)=-2.212, p=0.037) (Table 3).

**Table 3:** Comparison of energy needs between COVID-19 patients who had and those who did not have PTE: results of the t-test for two independent samples *Statistically significant differences at a 5% significance level REE, resting energy expenditure; EEABW, energy expenditure by adjusted body weight; EEKg, energy expenditure by actual weight; RQ, respiratory quotient; PTE, pulmonary thromboembolism

	PTE	Group statistics				Test statistics		
		N	Mean	Std. deviation	Std. error mean	t-Value	df	p-Value
REE	No	26	1649.115	392.995	77.072	-1.686	20.490	0.107
	Yes	8	1838.375	230.835	81.612			
EEABW	No	26	21.625	3.871	0.759	-0.994	32	0.328
	Yes	8	23.450	6.386	2.258			
EEKg	No	26	20.000	4.098	0.803	-0.899	32	0.375
	Yes	8	21.750	6.777	2.396			
RQ	No	26	0.896	0.085	0.01660	-2.212	32	0.034*
	Yes	8	0.970	0.077	0.02719			

## Discussion

This study compared the REE, energy expenditure by adjusted body weight (EEABW), energy expenditure by actual weight (EEKg), and RQ between COVID-19 and non-COVID-19 critically ill patients' metabolic profiles.

No statistically significant differences were observed in REE, EEABW, or EEKg, regardless of COVID-19 status. This finding suggests that the underlying pathophysiology of COVID-19 may not substantially alter overall energy requirements compared to other critical illnesses. However, it is important to note that while not statistically significant, there was a trend towards lower energy needs in COVID-19 patients. This trend aligns with observations that reported lower than expected EE in COVID-19 patients, potentially due to reduced physical activity and the effects of sedation and neuromuscular blockade commonly used in these patients [8].

Regarding RQ, a significantly higher RQ was found in COVID-19 patients [10]. A higher RQ suggests a metabolic shift towards carbohydrate use for energy production [7,11]. Several factors associated with COVID-19 may contribute to this observation, such as insulin resistance, suppression of fatty acid oxidation, and corticosteroid use. Acute insulin resistance and hyperglycemia [12] are associated with an increased reliance on carbohydrate metabolism, resulting in a higher RQ [7,11]. The systemic inflammation, characteristic of severe COVID-19, may suppress fatty acid oxidation, further shifting metabolism towards carbohydrate use [11-15]. Treatment of COVID-19 still requires the use of corticosteroids [16], as seen in our COVID-19 cohort. This could contribute to altered substrate metabolism, as these drugs are known to promote protein catabolism and potentially influence carbohydrate metabolism [17]. The clinical implications of this metabolic shift warrant further investigation, as it may suggest a need for careful glucose management and potentially tailored nutritional strategies that account for altered substrate use in COVID-19 patients [14,15].

Pertaining to the occurrence of PTE, within the COVID-19 group, patients without PTE exhibited a significantly lower RQ compared to those with PTE. This finding suggests a potential link between PTE and altered substrate use. Patients without PTE might favor fat metabolism, possibly due to a less severe illness and as such a lower impact on gas exchange. Consequently, they may exhibit a preference for using fats as an energy source compared to carbohydrates [18]. However, differences in pulmonary perfusion due to small vessel occlusion associated with PTE may affect IC measurements rather than reflecting true metabolic changes. Further investigation with additional metabolic assessments is needed to elucidate this relationship.

In the entirety of the population studied (COVID-19 and non-COVID-19 patients), factors such as BMI, ventilation mode (PA), and muscle blockade emerged as significant predictors of energy needs. These findings align with clinical expectations, as increased BMI [19], MV support [20], and muscle paralysis [21] are all known to contribute to higher EE.

This study has several limitations, regarding the relatively small sample, particularly in the COVID-19 group, which limits statistical power and may have precluded detection of smaller but clinically relevant differences. The retrospective design of the study increases the risk of confounding factors and limits causal inferences, and data from a single institution may limit generalizability to other settings or patient populations. The relatively small sample size, particularly in the COVID-19 group, limits the statistical power of the study. Future research directions should include prospective studies with larger, multi-center cohorts to confirm and expand upon these findings along with longitudinal assessments to track metabolic changes throughout the course of COVID-19 illness and recovery.

Despite the lack of statistically significant differences in energy needs, the observed trends and the association between RQ and COVID-19 status highlight the importance of considering a patient's specific metabolic profile when determining nutritional support. Tailoring nutritional strategies to account for potential fuel source preferences could optimize patient outcomes. Furthermore, understanding the link between PTE and RQ in COVID-19 patients warrants further investigation, potentially leading to personalized treatment approaches based on metabolic variations.

## Conclusions

Despite a lack of statistically significant differences in energy needs, the study highlights the importance of considering individual metabolic profiles in nutritional support for critically ill patients. The observed trends suggest potential fuel source preferences that could optimize patient outcomes. Additionally, the association between PTE and RQ in COVID-19 patients warrants further exploration, potentially leading to personalized metabolic-based treatment approaches.
